# Effectiveness of Endoscope‐Assisted Subgingival Debridement Versus Repeated Root Surface Debridement or Access Flap Periodontal Surgery in Step 3 Periodontal Therapy: A Systematic Review and Meta‐Analysis

**DOI:** 10.1002/cre2.70196

**Published:** 2025-07-31

**Authors:** King‐Lun Dominic Ho, Melissa Rachel Fok, Kar Yan Li, Georgios Pelekos, Wai Keung Leung

**Affiliations:** ^1^ Periodontology and Implant Dentistry, Faculty of Dentistry the University of Hong Kong Hong Kong SAR China; ^2^ Clinical Research Centre, Faculty of Dentistry the University of Hong Kong Hong Kong SAR China

**Keywords:** endoscopes, meta‐analysis, periodontal debridement, periodontitis, systematic review

## Abstract

**Objectives:**

Periodontitis is a multifactorial inflammatory disease leading to the progressive destruction of the tooth‐supporting apparatus. The management of residual periodontal pockets remains a challenge for Step 3 periodontal therapy. This systematic review aims to evaluate the potential and efficacy of the periodontal endoscope in managing residual periodontal pockets during Step 3 periodontal therapy.

**Material and Methods:**

A comprehensive search was conducted in Medline, PubMed, Cochrane Library, Embase, Scopus, and Web of Science databases up to December 2024. Studies included were randomized controlled trials (RCTs) comparing periodontal endoscope‐assisted subgingival debridement (EASD) with repeated root surface debridement (RSD) and access flap periodontal surgery (AFPS). Data extraction and risk of bias assessment were performed independently by two reviewers.

**Results:**

Five RCTs were included, involving 155 subjects and 4072 sites. EASD showed a significantly higher periodontal probing depth (PPD) reduction compared to repeated RSD, with a weighted mean difference (WMD) of 0.5 mm (95% CI: 0.19–0.81) at 3‐month postoperation. At 6‐month postoperation, the WMD of PPD and clinical attachment level (CAL) changes were 0.84 mm (95% CI: 0.60–1.09) and 0.89 mm (95% CI: 0.45–1.34), respectively, in favor of EASD. EASD showed a significantly higher prevalence ratio (20%) of pocket resolution (PPD ≤ 4 mm) compared to repeated RSD at 6‐month postoperation. No significant differences were observed between EASD and AFPS in the changes of CAL, PPD and prevalence of pocket resolution (PPD ≤ 4 mm). The overall certainty of the evidence was deemed to be “low” for EASD versus repeated RSD comparisons and “moderate” for EASD versus AFPS comparisons.

**Conclusions:**

EASD demonstrated superior clinical outcomes compared to repeated RSD in managing residual periodontal pockets. Further high‐quality research is necessary to validate these findings and explore the long‐term benefits of EASD.

## Summary

- Endoscope‐assisted subgingival debridement (EASD) improves clinical attachment level (CAL) at 6‐month, and probing pocket depth (PPD) at 3‐ and 6‐month postoperation compared to repeated root surface debridement (RSD).
- No significant difference between EASD and access flap periodontal surgery (AFPS). in CAL gain, PPD reduction and prevalence of pocket resolution at 3 months postoperation.
- Higher prevalence ratio (20%) on pocket resolution (PPD ≤ 4 mm) in favor of EASD over repeated RSD was observed at 6 months postoperation.

## 1 Introduction

Periodontitis is a complex inflammatory disease that causes the progressive destruction of the structures supporting the teeth, including alveolar bone, periodontal ligament, and cementum. As the disease advances, it results in loss of periodontal support, leading to impairments in speech, chewing, esthetics, occlusal stability, and ultimately tooth loss. Effective management hinges on controlling the dysbiotic dental biofilm, primarily through mechanical debridement of the root surfaces, which creates a biologically acceptable environment that promotes healing and resolution of inflammation (Pelekos et al. 2023).

Recent clinical practice guidelines (CPG) for periodontitis advocate a four‐step, incremental treatment approach tailored to disease severity and patient risk factors (Sanz et al. 2020). The initial steps (Steps 1 and 2) involve patient education, motivation, risk factor management, and nonsurgical periodontal therapy (NSPT), which includes scaling and root planing. These procedures aim to disrupt pathogenic biofilms, reduce pocket depths, and resolve inflammation, often leading to pocket resolution and clinical attachment gain.

Posttreatment re‐evaluation occurs after adequate healing time (Claffey 1991; Lang and Tonetti 1996). The goal is to achieve periodontal pockets of less than 5 mm with no Bleeding on Probing (BoP), thereby facilitating maintenance therapy (Step 4) (Sanz et al. 2020). However, individual sites may respond differently due to local and systemic factors, such as deep probing depths ( > 6 mm), root surface anatomy (concavities, furcations), and intrabony defects, which can limit the success of NSPT.

Residual periodontal pockets with persistent deep probing depths often necessitate additional intervention, termed Step 3 therapy (Sanz et al. 2020). For sites with moderate periodontal probing depth (PPD) (4–6 mm), repeated subgingival instrumentation is recommended. Deeper pockets ( ≥ 6 mm) may require surgical intervention, such as an access flap surgery, to enhance visibility and improve the efficacy of debridement.

Access flap surgery, including resective procedures, has historically aimed to improve access for thorough root debridement and the correction of defects, such as furcations or shallow intrabony lesions (Kirkland 1947; O. 1931; Ramfjord and Nissle 1974). These procedures can reduce pocket depths but may cause gingival recession, loss of attached tissue, and compromise esthetics. Recognizing the importance of alveolar bone and soft tissue preservation, more conservative access flap surgical techniques were developed, such as the modified Widman flap, which minimizes bone removal and aims to maintain the periodontal architecture. Despite these advances, studies have shown that intrabony defects often persist even after flap surgery, indicating limited predictability of defect resolution solely through resective approaches (Dommisch et al. 2020; Kaldahl et al. 1988; Kaldahl et al. 1996; Ramfjord et al. 1987). For example, research suggests that most intrabony defects remain after traditional surgical treatment, with only partial improvements in pocket depths and attachment levels.

In contrast to resective procedures, regenerative techniques seek to restore lost periodontal structures. guided tissue regeneration (GTR) utilizes barrier membranes and bioactive materials to facilitate the formation of new attachment. Meta‐analyses demonstrate that regenerative procedures can produce additional gains in clinical attachment and pocket reduction compared to open flap debridement alone, making them valuable in managing intrabony defects.

While surgical options are effective, nonsurgical management of intrabony defects remains an area of active investigation. Some studies suggest that repeated nonsurgical therapy can lead to clinical improvements, including reductions in pocket depth and gains in attachment, especially when combined with adjunctive technologies (Badersten et al. 1981, 1984, 1985a, 1985b, 1985c; Nibali et al. 2011). Notwithstanding the fact that in some clinical situations, the extent of improvement may not be as much when compared to surgical periodontal therapy (Renvert et al. 1985). The emergence of minimally invasive nonsurgical approaches, involving magnification and careful root debridement, has shown promising long‐term results, with significant reductions in probing depths and defect depths, as well as stable outcomes over a 5‐year period (Nibali et al. 2015; Nibali et al. 2018).

A notable innovation is the use of periodontal endoscopes, which enhance visualization of subgingival surfaces during NSPT (Stambaugh et al. 2002). These devices enable clinicians to identify residual calculus and plaque that may be missed with traditional tools, potentially enhancing debridement quality in challenging‐to‐access areas, such as deep intrabony defects (Geisinger et al. 2007; Michaud et al. 2007).

However, clinical evidence regarding the benefits of periodontal endoscope‐assisted subgingival debridement (EASD) is mixed (Avradopoulos et al. 2004; Blue et al. 2013; Graetz et al. 2022). Some studies reported no significant additional clinical benefit, while others demonstrate improved outcomes, including greater pocket depth reductions, attachment gains, and reduced residual calculus, particularly in deep pockets (Liao et al. 2016; Naicker et al. 2022; Wright et al. 2023; Wu et al. 2022; Xu et al. 2021; Zhang et al. 2020). The heterogeneity of study designs, defect morphologies, and clinical scenarios complicates the drawing of definitive conclusions.

Systematic reviews on periodontal endoscopy present conflicting results (Ardila and Vivares‐Builes 2023; Kuang et al. 2017). One highlighted some clinical benefits, such as lower residual calculus, but often at the expense of increased chairside time. The evidence to suggest additional benefit on the improvement of clinical parameters is insufficient (Kuang et al. 2017). Significant clinical advantages in terms of PPD reduction, improvement on clinical attachment level (CAL) and BoP over traditional NSPT were concluded in another review (Ardila and Vivares‐Builes 2023). This inconsistency is partly due to the heterogeneity of study populations, defect types, treatment protocols, and the lack of high‐quality randomized controlled trials (RCTs) for meta‐analysis.

One key challenge is effectively managing deep, narrow intrabony defects during nonsurgical therapy. While technological advances such as magnification, specialized instruments, and endoscopes enhance visualization, operator skill remains crucial. The tactile feedback and limited access to certain defects remain persistent obstacles.

Research is ongoing to clarify the role of periodontal endoscopy in routine periodontal therapy. Recent studies suggest that with proper case selection, especially in deep or residual intrabony defects, adjunctive use of endoscopes may enhance debridement, potentially improving long‐term outcomes (Ho et al. 2025). Nevertheless, high‐quality RCTs are necessary to establish standardized protocols and confirm the actual benefits of these technologies.

Despite the growing number of investigations demonstrating positive results on the application of periodontal endoscopes to assist repeated root surface debridement (RSD) in Step 3 periodontal therapy in non‐English medium, considerable heterogeneity and controversy remain. Determining the real therapeutic value of the periodontal endoscope in managing residual periodontal pockets is essential. Therefore, the aim of this systematic review and meta‐analysis is to evaluate the potential and efficacy of the periodontal endoscope in the management of residual periodontal pockets in Step 3 periodontal therapy.

### 1.1 Aims and Objectives

- To compare the clinical outcome variables among different applications of periodontal endoscopes in the treatment of residual periodontal pockets in Step 3 periodontal therapy.
- To systematically review the evidence documenting the use of periodontal endoscopes in the management of residual periodontal pockets concerning clinical, radiographic, and patient‐centered outcomes.

## 2 Materials and Methods

The current systematic review was written and reported according to the PRISMA guideline (Haddaway et al. 2022). The protocol was registered in the Open Science Framework database, hosted by the Center for Open Science, on December 12, 2024. The protocol can be accessed publicly via this hyperlink: https://osf.io/2epk5. This study was substantially supported by the Health and Medical Research Fund of Hong Kong (grant No. 18170492).

### 2.1 Focused Question

The focus question of this review is as follows: “Does the application of periodontal endoscope in the management of residual periodontal pockets provide additional clinical benefits, in periodontitis patients, when compared to the periodontal therapies without the assistance of periodontal endoscope in Step 3 periodontal therapy?”

### 2.2 Eligibility Criteria

Criteria, based on the following PICOS method, was adopted for this systematic review for the inclusion of studies.

1. Population (P): Adult patients (aged ≥ 18 years) who presented with periodontitis (Caton et al. 2018) and received initial periodontal therapy (Steps 1 and 2 periodontal therapy), showing at least one residual periodontal pocket(s) with PPD and CAL 5 mm, after a minimum of 6 weeks of healing.
2. Intervention (I): Step 3 periodontal therapy, including the following procedure, along with the application of a periodontal endoscope
  - Repeated subgingival instrumentation
  - Access flap periodontal surgery (AFPS)
  - Regenerative periodontal surgery
3. Comparison (C): Step 3 periodontal therapy, including the following procedures, without adjunctive application of periodontal endoscope
  - Repeated subgingival instrumentation
  - AFPS
  - Regenerative periodontal surgery
4. Outcome (O): The primary outcomes include the change in CAL. The secondary outcomes included change in PPD, REC, and pocket resolution (PPD ≤ 4 mm at study follow‐up).

#### 2.2.1 Inclusion Criteria

This review included human randomized controlled clinical trials (RCTs) with parallel or split‐mouth designs, comparing at least two of the investigated techniques, with a minimum of 10 sites per arm and a minimum follow‐up period of 3 months.

#### 2.2.2 Exclusion Criteria

Abstracts, protocols, book chapters, proceedings, case reports, reviews, nonhuman studies, and those without valid control were excluded.

### 2.3 Information Sources and Search Strategy

A reviewer (D.K.L.H.) searched electronic databases until December 4, 2024, to identify studies for this systematic review using the following information sources: Medline via Ovid, National Library of Medicine via PubMed, The Cochrane Library, Embase, Scopus, and Web of Science, with the following search statement.

(“periodontal endoscope” OR “subgingival endoscope” OR “periodontal microsurgery” OR “Videoscop*” OR “endoscop*”) AND (“periodontitis” OR “chronic periodontitis” OR “periodontal disease” OR “Periodont*”)

The hand search included a comprehensive review of the *Journal of Clinical Periodontology* (JCP), *Journal of Periodontology* (JP), *Journal of Periodontal Research* (JPR), and *Journal of Dental Research* (JDR) up to December 2024. JCP and JP provided an additional 37 and 9 entries, respectively, but these did not yield any further data. JPR and JDR did not offer any entries. The hand search was complemented by screening the reference lists of previous systematic reviews related to the application of periodontal endoscopes; however, all included articles were already part of the previous electronic search. Additionally, a gray literature search using the Health Management Information Centre via Ovid in the field of periodontal endoscopy did not provide any additional data. The same experience was observed via SCOPUS when searching for articles that cited the included papers.

### 2.4 Selection Process

The search results including the references, journal titles, study titles, authors, years of publication, abstracts and relevant information were exported to the Covidence platform (Covidence systematic review software, Veritas Health Innovation, Melbourne, Australia, www.covidence.org). Duplications were removed by the built‐in detection program before constructing the final list for review selection.

The review selection consisted of two steps. The first step involved screening by two independent reviewers (D.K.L.H. and M.R.F.) to assess the title and abstract based on the predetermined inclusion and exclusion criteria. If there is insufficient information to evaluate the criteria, full articles will be obtained. Any conflicts in decisions regarding inclusion between the two reviewers were resolved by the third independent reviewer (M.N.T.).

The second step involved screening by appraising the full text using the table of eligibility criteria. All steps were performed independently by two reviewers (D.K.L.H. and M.R.F.). Any disparity was resolved through open discussion with the third independent reviewer (M.N.T.) until a consensus was achieved. Ineligible articles were excluded with reasons documented.

### 2.5 Data Collection Process and Data Items

All included studies, following full‐text screening, were assessed on their quality and risk of bias, and data extraction was conducted by two independent assessors (D.K.L.H. and M.R.F.). Data extraction was conducted using a structured EXCEL form. The data included the number of subjects, demographic information, types of intervention, observation period, mean and standard deviation of clinical outcomes related to PPD, CAL at baseline and the percentage of subjects (sites) achieving pocket resolution postoperation at different time points. Any changes in clinical data were recorded if reported in the study, or the estimated change was computed by the biostatistician (K.Y.L.) if not reported. Disagreements on the extracted data were resolved through consensus between two assessors and discussion with the biostatistician.

### 2.6 Risk of Bias Assessment

The quality and risk of bias of the included studies were independently assessed by the two review authors (D.K.L.H. and M.R.F.) using separate electronic forms. The Cochrane risk‐of‐bias tool for randomized trials (RoB2) was used for the assessment exercise (Sterne et al. 2019). Risks of bias arising from the randomization process, deviations from the intended interventions (effect of assignment and adherence to intervention), missing outcome data, outcome measurement, and selection of the reported results were assessed. Any disagreement in the assessment was resolved through discussions between the two review authors. An overall risk of bias judgment was assigned to all included studies based on the following criteria.

Lower risk of bias: The study is judged to be at low risk of bias for all domains for this result.
Some concerns: The study is judged to raise some concerns in at least one domain for this result, but not to be at high risk of bias for any domain.
High risk of bias: The study is judged to be at high risk of bias in at least one domain for this result, or the study is judged to have some concerns for multiple domains in a way that substantially lowers confidence in the result.

### 2.7 Effect Measures

Effect measures were measured in mean difference for comparisons in all continuous outcomes, including change in CAL, PPD, and REC, while measured in prevalence ratio for the comparison in the prevalence of sites undergoing pocket resolution (PPD ≤ 4 mm at study follow‐up).

### 2.8 Synthesis Methods

The included studies were summarized narratively according to their interventions and outcomes. Random‐effect meta‐analysis was performed using the DerSimonian and Laird (D.L.) method if at least two included studies with similar study designs reported the same field of data. Random‐effect approach is chosen to account for potential heterogeneity across studies and to facilitate generalization of the findings. The mean and standard deviation of the CAL, PPD, and REC at different time points and their changes and percentage of sites undergone pocket resolution (PPD ≤ 4 mm) were extracted from the original publication by two reviewers (D.H. and M.F.) and compared between treatment arms. Disagreement was solved by discussion. If the required outcomes were not reported in the publication, estimated value of the data was computed based on the reported outcome measures whenever possible. Continuous data, CAL, PPD, and REC were presented as mean difference with 95% confidence interval. Percentage of sites undergoing pocket resolution was presented as a prevalence ratio with 95% confidence intervals. Four out of the five included studies were designed as cluster RCTs, with multiple sites per patient introducing within‐patient clustering. To account for within‐cluster correlation, standard errors were inflated based on the design effect, which was calculated from the average cluster size and Intracluster Correlation Coefficient (ICC) recommended by the Cochrane handbook. ICC was set as 0.4 based on estimates from a previous dental study (Meinhold et al. 2020). A sensitivity analysis using an ICC of 0.2 was conducted to assess the robustness of results to clustering assumptions (Wan et al. 2009). The heterogeneity assessment was conducted with a Chi‐square‐based Q‐statistic approach and the I‐squared metric. Heterogeneity was considered as significant if the I‐square value exceeded 40% or a *p*‐value for the Chi‐squared test below 0.10. We intended to assess nonreporting bias using funnel plot techniques, Begg's rank test, and Egger's regression test, as appropriate given the known limitations of these methods if the number of studies is ten or larger recommended by Cochrane handbook. Meta‐analysis was conducted with the statistical software packet Stata version 16 (StataCorp, College Station, TX).

### 2.9 Certainty Assessment

The certainty assessment of the evidence was evaluated following the GRADE Criteria, including types of study design, risk of bias, inconsistency, indirectness, imprecision, and publication bias. The exercise of assessment was conducted with the GRADEpro software program (GRADEpro guideline Development Tool software, McMaster University and Evidence Prime 2021, www.gradepro.org).

## 3 Result

### 3.1 Study Selection

**Figure 1:**
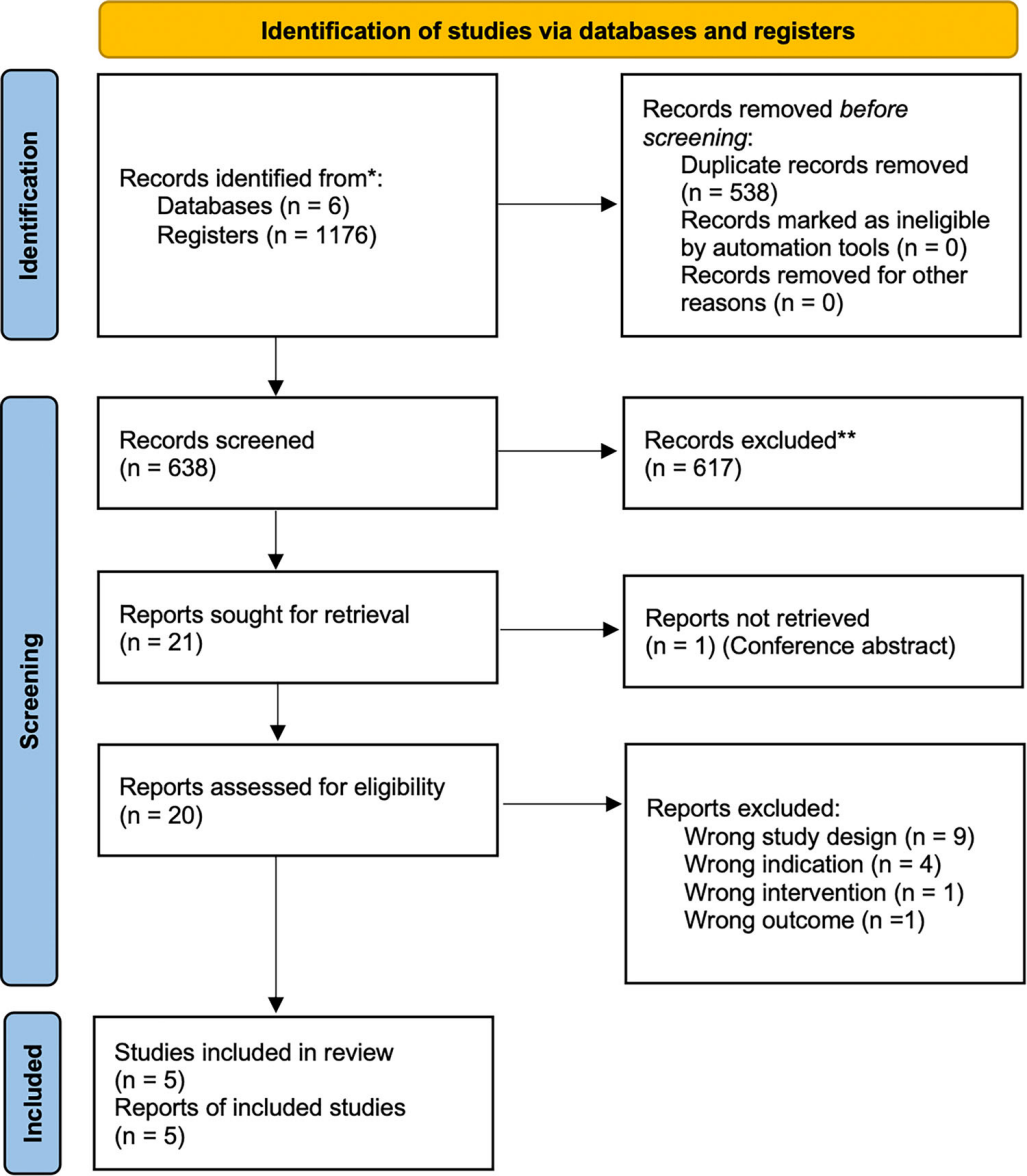
Flow diagram.

The search result is presented in Figure 1. The electronic search was performed using Medline via Ovid, National Library of Medicine via PubMed, The Cochrane Library, Embase, Scopus, and Web of Science, resulting in 1176 articles published until December 4, 2024. After removing 538 duplicated records, 638 records entered the title and abstract screening stage. Of these, 617 were found ineligible for inclusion. Full text of one record (conference abstract) could not be retrieved. The full text of the 20 eligible studies was obtained for assessment against the inclusion and exclusion criteria. 15 articles were excluded (Table S1) and 5 articles were included for data extraction (Table 1). The hand search process found zero additional articles.

**Table 1:** Summary of the included studies.

		Zhang et al. (2020)*	Xu et al. (2021)	Wu et al. (2022)*	Pei et al. (2023)	Ho et al. (2025)
**Study design**		RCT, Parallel group	RCT, Split‐mouth	RCT, Parallel group	Site‐level RCT	RCT, Parallel group
**Location**		Nanjing, China, Hospital	Chengdu, China, Hospital	Nanjing, China, Hospital	Beijing, China, Hospital	Hong Kong, China, Hospital
**Interventions**	Test vs. Control	EASD vs. RSD	EASD vs. RSD	EASD vs. RSD	EASD vs. AFPS (Open Flap Debridement)	EASD vs. AFPS (Papilla Preservation Flap Surgery)
**Number of subjects recruited**	*n*	40	13	40	5	64
**Number of subjects evaluated**	Test/Control	20/18	13/13	19/18	5 in total	30/32
**Number of sites evaluated**	Test/Control	851/795	337/357	830/799	21/20	30/32
**Age (years)**	Mean (SD)	Test: 36.6 ± 8.1; Control: 34.4 ± 8.2	35.9 ± 7.1	Test: 37.6 ± 7.8; Control: 35.6 ± 8.1	40.0 ± 10.1	Test: 57.0 ± 11.1; Control: 57.5 ± 11.5
**Site characteristics**		Residual periodontal defect with PPD ≥ 5 mm	Residual periodontal defect with PPD ≥ 4 mm with BoP CAL ≥ 4 mm	Residual periodontal defect with PPD ≥ 5 mm	Residual periodontal defect with PPD ≥ 5 mm with BoP CAL > 3 mm	Residual periodontal defect with PPD ≥ 5 mm with BoP or ≥ 6 mm CAL > 6 mm intrabony defect ≥ 3 mm
**Follow‐up period (months)**		6	3	6	3	12
**Change of CAL (Test) (mm)**		3‐month: 1.17 ± 1.98 6‐month: 1.73 ± 1.97	3‐month: 1.14 ± 1.48 (Single‐rooted) 1.14 ± 1.38 (Multi‐rooted)	3‐month: 1.71 ± 0.84 6‐month: 2.01 ± 0.84	3‐month: 0.36 ± 1.20	3‐month: 1.90 ± 1.25 6‐month: 1.90 ± 1.21 9‐month: 1.90 ± 1.30 12‐month: 2.3 ± 0.90
**Change of CAL (Control) (mm)**		3‐month: 1.17 ± 2.08 6‐month: 1.03 ± 2.08	3‐month: 0.99 ± 1.37 (Single‐rooted) 1.08 ± 1.33 (Multi‐rooted)	3‐month: 1.30 ± 0.88 6‐month: 1.28 ± 0.88	3‐month: 0.5 ± 1.43	3‐month: 1.60 ± 1.71 6‐month: 1.50 ± 1.48 9‐month: 1.70 ± 1.39 12‐month: 2.1 ± 1.00
**Change of PPD (Test) (mm)**		3‐month: 2.45 ± 1.07 6‐month: 2.88 ± 1.07	3‐month: 1.84 ± 0.89 (Single‐rooted) 1.48 ± 0.85 (Multi‐rooted)	3‐month: 2.6 ± 0.38 6‐month: 2.93 ± 0.38	3‐month: 1.67 ± 0.91	3‐month: 2.37 ± 1.13 6‐month: 2.45 ± 1.09 9‐month: 2.61 ± 0.99 12‐month: 2.68 ± 1.02
**Change of PPD (Control) (mm)**		3‐month: 2.07 ± 1.06 6‐month: 2.14 ± 1.06	3‐month: 1.38 ± 0.79 (Single‐rooted) 1.25 ± 0.80 (Multi‐rooted)	3‐month: 1.66 ± 0.31 6‐month: 1.80 ± 0.31	3‐month: 2.25 ± 1.12	3‐month: 2.06 ± 0.84 6‐month: 2.19 ± 0.87 9‐month: 2.31 ± 0.97 12‐month: 2.38 ± 0.94
**Change of REC (Test) (mm)**		NR	NR	NR	NR	3‐month: −0.23 ± 0.63 6‐month: −0.31 ± 0.54 9‐month: −0.32 ± 0.67 12‐month: −0.36 ± 0.62
**Change of REC (Control) (mm)**		NR	NR	NR	NR	3‐month: −0.38 ± 0.66 6‐month: −0.35 ± 0.61 9‐month: −0.31 ± 0.78 12‐month: −0.44 ± 0.56
**Pocket closure (Test) (%)**		3‐month: 79.9 6‐month: 86.2	NR	3‐month: 82.05 6‐month: 87.59	3‐month: 76.00	3‐month: 93.30 6‐month: 86.70 9‐month: 93.30 12‐month: 86.70
**Pocket closure (Control) (%)**		3‐month: 73.2 6‐month: 72.8	NR	3‐month: 68.46 6‐month: 71.59	3‐month: 85.00	3‐month: 87.50 6‐month: 93.80 9‐month: 90.60 12‐month: 90.60
**Funding**		Public	Public	Public	Public	Public

Abbreviations: AFPS, access flap periodontal surgery; BoP, bleeding on probing; CAL, clinical attachment level; EASD, endoscope‐assisted subgingival debridement; NR, not reported; PPD, periodontal pocket depth; RSD, root surface debridement; RCT, randomized clinical control trial.

*Shared same last author; supported by multiple grants, including one supporting both reports; same human ethics approval number; reported studies took place over similar period ‐ April 2016‐Februray 2019 (Zhang et al. 2020) or October 2017 to December 2019 (Wu et al. 2022); exact male/female count in RSD group of 8/10 and exact dropout count but different mean age reported; nil same data reported from either paper.

### 3.2 Study Characteristics

The five included studies were all university‐ or public‐hospital‐based randomized clinical controlled trials and were supported by public funding. Four studies were conducted in China, and one study was conducted in Hong Kong, China. Four studies were conducted in a parallel group design, and one was a split‐mouth design. One of the studies reported recruitment of 41 sites (21 EASD, 20 AFPS, no indication of site location) from five patients lacking an explicit study design (Pei et al. 2023). Another study recruited only one site from each subject (Ho et al. 2025), while the rest of the studies were cluster‐design and reported treatment outcomes from multiple sites from each subject (Pei et al. 2023; Wu et al. 2022; Xu et al. 2021; Zhang et al. 2020). Two studies were published by the same research group within a similar time frame (Wu et al. 2022; Zhang et al. 2020). Three studies compared EASD versus repeated RSD (Wu et al. 2022; Xu et al. 2021; Zhang et al. 2020). Two studies compared EASD versus AFPS, including open flap debridement and papilla preservation flap surgery (Ho et al. 2025; Pei et al. 2023). A total of 155 subjects with 4072 sites (EASD: 2069; AFPS: 52; repeated RSD: 1951) were included for analysis. Two studies reported outcomes up to 3 months after intervention (Pei et al. 2023; Xu et al. 2021), while two studies reported up to 6 months after intervention (Wu et al. 2022; Zhang et al. 2020). Only one study reported the clinical outcomes up to 12 months after the operation (Ho et al. 2025). The summary of the included studies with outcome variables is presented in Table 1.

### 3.3 Quality Assessment

The quality of the included studies was assessed with the Cochrane risk‐of‐bias tool for randomized trials (RoB2) (Figure S1). Three out of five studies were graded with high risk of bias (Wu et al. 2022; Xu et al. 2021; Zhang et al. 2020), and two had some concerns on the risk of bias (Ho et al. 2025; Pei et al. 2023). In four out of five included studies, details regarding breakage of concealment were not reported, leading to some concerns being raised for the domain of randomization (Pei et al. 2023; Wu et al. 2022; Xu et al. 2021; Zhang et al. 2020). In two of the included studies, one study reported that three subjects were excluded from the analysis, in which one was lost during follow‐up and two required periodontal surgical intervention. The other study reported two subjects excluded from analysis – one due to loss in follow‐up and one requiring surgical intervention. Therefore, both studies were given a high risk of bias in Domain 2 (deviation from the intended interventions) (Wu et al. 2022; Zhang et al. 2020). The pre‐specific analysis plans of all studies were not retrievable from public registration databases, resulting in four included studies being graded with some concerns in the selection of the reported result domain (Ho et al. 2025; Pei et al. 2023; Wu et al. 2022; Zhang et al. 2020). One study conducted multiple analyses of clinical data with subgroup analysis based on the time of healing and tooth type, leading to a high risk of concern in this domain (Xu et al. 2021).

### 3.4 Result of the Analysis

The five included studies had various observation periods ranging from 3 months to 12 months. Comparisons of the change in CAL, PPD, and percentage of sites with resolution of pockets between EASD versus repeated RSD and EASD versus AFPS were made at different time points, with at least 2 available studies in each comparison. However, only one study reported the change of REC, and thus no meta‐analysis could be conducted for this outcome.

### 3.5 Clinical Attachment Level

**Figure 2:**
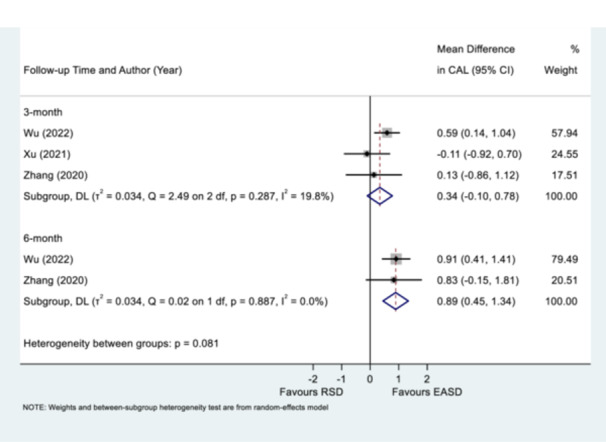
Mean difference of clinical attachment level (CAL) 3‐ or 6‐month posttreatment of endoscope‐assisted subgingival debridement (EASD) or repeated root surface debridement (RSD). Please note Zhang et al. (2020) and Wu et al. (2022) shared same last author; supported by multiple grants, including one supporting both reports; same human ethics approval number; reported studies took place over similar period – April 2016–Februray 2019 (Zhang et al. 2020) or October 2017 to December 2019 (Wu et al. 2022); exact male/female count in RSD group of 8/10 and exact dropout count but different mean age reported; nil same data reported from either paper.

The meta‐analysis of the change of CAL between EASD and repeated RSD, at the 3‐month interval, indicated no statistically significant difference between the interventions, with a weighted mean difference (WMD) of 0.34 mm (95% CI: −0.10 to 0.78) and low heterogeneity (Q = 2.49 on 2 df, *p* = 0.287, I^2^ = 19.8%). Conversely, the 6‐month follow‐up subgroup revealed a statistically significant improvement in CAL favoring EASD, with a WMD of 0.89 mm (95% CI: 0.45–1.34) and no observed heterogeneity (Q = 0.02 on 1 df, *p* = 0.887, I^2^ = 0.0%). The test for subgroup differences showed a *p*‐value of 0.081, suggesting no time‐dependent effect of the interventions (Figure 2).

Regarding the comparison between EASD and APFS, it showed a WMD of 0.21 mm (95% CI: −0.49 to 0.92) in favor of EASD, but no statistically significant difference was detected. The analysis exhibited low heterogeneity between the studies (Q = 0.17 on 1df, *p* = 0.680, I^2^ = 0.0%) (Figure S2).

### 3.6 Periodontal Probing Depth

**Figure 3:**
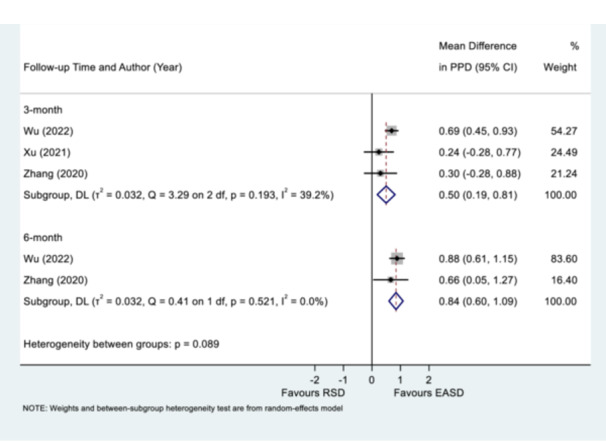
Mean difference of probing pocket depth (PPD) 3‐ or 6‐month posttreatment of endoscope‐assisted subgingival debridement (EASD) or repeated root surface debridement (RSD). Please refer to Table 1 for data concerning Zhang et al. (2020) and Wu et al. (2022).

The comparison of the change in PPD between EASD and repeated RSD at the 3‐month follow‐up indicated a statistically significant reduction favoring EASD, with a WMD of 0.50 mm (95% CI: 0.19–0.81), and demonstrated marginally low heterogeneity (Q = 3.28 on 2 df, *p* = 0.194, I² = 39.1%). Similarly, the 6‐month follow‐up subgroup also demonstrated a statistically significant improvement in PPD for the EASD group, with a WMD of 0.84 mm (95% CI: 0.60–1.09) and no observed heterogeneity (Q = 0.41 on 1 df, *p* = 0.521, I² = 0.0%). The test for heterogeneity between subgroups indicated a *p*‐value of 0.090, suggesting no significant difference in the effect of the interventions over time (Figure 3).

The meta‐analysis of the change in PPD between EASD and APFS indicated no statistically significant difference between the two interventions. The overall result from the random‐effects model showed a WMD of −0.29 mm (95% CI: −0.69 to 0.11). An analysis of heterogeneity found no variance between the study results (Q = 0.02 on 1 df, *p* = 0.888, I² = 0.0%) (Figure S3).

### 3.7 Gingival Recession

Only one study reported the change of gingival recession over the 12‐month observational period; therefore, no meta‐analysis was performed. In the included studies comparing the EASD versus AFPS with papilla preservation flap surgery, only a trend of minor changes of gingival recession in favor of AFPS was observed over the 12‐month healing period (Figure S4).

### 3.8 Pocket Resolution

**Figure 4:**
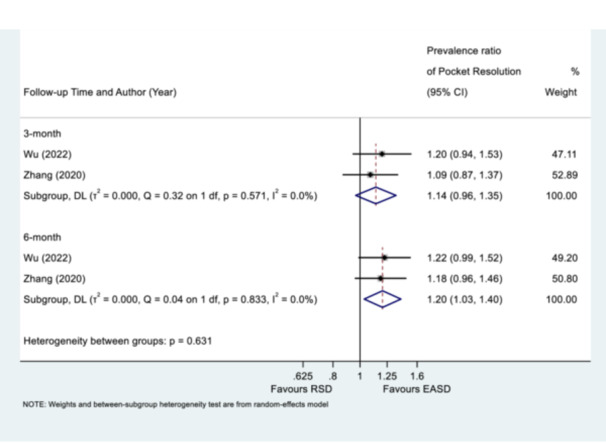
Prevalence ratio of pocket resolution (PPD ≤ 4 mm) 3‐ or 6‐month posttreatment of endoscope‐assisted subgingival debridement (EASD) or repeated root surface debridement (RSD). Please refer to comments on Figure 3 or Table 1 concerning Zhang et al. (2020) and Wu et al. (2022).

At 3 months, the pooled prevalence ratio for pocket resolution (PPD ≤ 4 mm) was 1.14 (95% CI: 0.96–1.35), indicating no statistically significant difference in prevalence of pocket resolution between EASD and repeated RSD (*p* = 0.126). No significant heterogeneity was observed within this subgroup (I^2^ = 0.0%, *p* = 0.571). However, at 6 months, EASD demonstrated a statistically significant higher prevalence of pocket resolution compared with repeated RSD, with a pooled prevalence ratio of 1.20 (95% CI: 1.03–1.40), in favor of EASD (*p* = 0.017). Similarly, no significant heterogeneity was detected within the 6‐month subgroup (I^2^ = 0.0%, *p* = 0.833). Analysis of heterogeneity between the 3‐month and 6‐month subgroups revealed no statistically significant difference in treatment effect across these time points (*p* = 0.631) (Figure 4). Conversely, in the comparison between EASD and APFS, the pooled prevalence ratio was calculated to be 1.05 (95% CI: 0.90–1.22) with no statistically significant difference (*p* = 0.566). Furthermore, the analysis revealed no significant statistical heterogeneity between the included studies (Q = 0.50 on 1 df, *p* = 0.477, I^2^ = 0.0%) (Figure S5).

### 3.9 Sensitivity Analysis

A sensitivity analysis with an ICC assumption of 0.2 showed that most of the results, except prevalence ratio of pocket resolution at 3 months in the comparison between EASD versus repeated RSD, remained unchanged. While, the prevalence ratio of pocket resolution at 3 months postoperation in the comparison between EASD versus repeated RSD changed from insignificant (1.14 [95% CI: 0.96–1.35] with *p* = 0.126 when ICC = 0.4) to statistically significant (1.14 [95% CI: 1.01–1.29] with *p* = 0.035 when ICC = 0.2), suggested that EASD might have a slightly higher likelihood of resolving pockets under this assumption. This indicated the 3‐month result was sensitive to changes in how study data were correlated.

### 3.10 Nonreporting Bias

Nonreporting bias was not assessed as there was an inadequate number of included trials (< 10) to properly assess a funnel plot or perform more advanced regression‐based assessments.

### 3.11 Certainty Assessment

**Figure 5:**
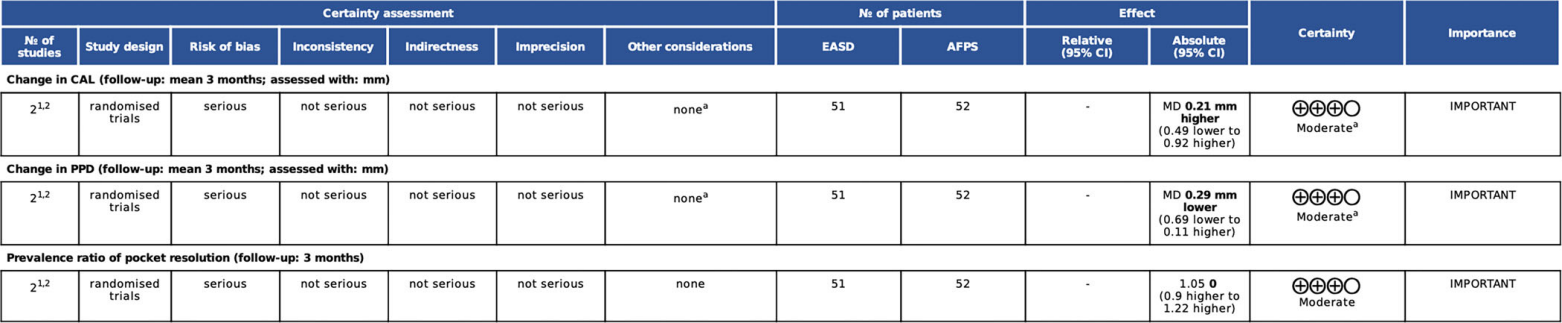
Grade assessment on evidence comparing endoscope‐assisted subgingival debridement (EASD) versus access flap periodontal surgery (AFPS).

The certainty of evidence was assessed using the GRADE approach. The certainty of the evidence for the difference in the change of PPD, CAL, and the prevalence ratio of pocket resolution between EASD and repeated RSD was rated as “low” (Figure S6). In contrast, the certainty of evidence for the change in PPD, CAL, and prevalence ratio of pocket resolution between EASD and AFPS was graded as “moderate” (Figure 5).

The GRADE system classifies the certainty of evidence in one of four grades:

**Table:** 

Grade	Definition
High	Further research is very unlikely to change our confidence in the estimate of effect.
Moderate	Further research is likely to have an important impact on our confidence in the estimate of effect and may change the estimate.
Low	Further research is very likely to have an important impact on our confidence in the estimate of effect and is likely to change the estimate.
Very low	Any estimate of effect is very uncertain.

## 4 Discussion

This is the first systematic review aimed at addressing the clinical efficacy of the application of periodontal EASD in a specific clinical scenario: whether the application of a periodontal EASD in the management of residual periodontal defects with increased PPD provides additional clinical benefits in Step 3 periodontal therapy. These potential benefits include gains in CAL, reductions in periodontal pocket depth, and changes in recession in periodontitis patients when compared to periodontal therapies without the assistance of a periodontal endoscope in Step 3 periodontal therapy. If the efficacy of the endoscopic approach is established, it has the potential to open up new avenues for reducing the need for surgical interventions in relevant procedures.

The application of the periodontal endoscope was first described in periodontal therapy to visualize the subgingival environment and confirm the relationship between subgingival deposits and periodontal inflammation (Wilson et al. 2008). This technology was used in conjunction with various treatment modalities and compared with existing treatment approaches. However, a systematic evaluation of the current evidence on the application of the periodontal endoscope is lacking, making it difficult to understand the true therapeutic value of this technology.

In the previous systematic reviews, Kuang et al. had evaluated 8 studies to compare the clinical application of periodontal endoscope in conjunction with RSD against traditional RSD (Kuang et al. 2017). It was reported that the application of periodontal endoscope‐assisted RSD resulted in a significantly less percentage of residual calculus on the root surface, but required longer treatment time to complete. The evidence to support the use of periodontal endoscopes as an adjunct to RSD was deemed as weak according to Kuang's systematic review (Kuang et al. 2017). In a more recent systematic review that included findings from 3 studies, it was reported that the periodontal endoscope‐assisted RSD showed significantly greater reductions in PPD, BOP, and plaque indices compared to repeated RSD alone. The review also noted some conflicting findings regarding the CAL (Ardila and Vivares‐Builes 2023). However, Ardila et al.'s systematic review did not conduct a meta‐analysis of the clinical data.

The two above‐discussed systematic reviews evaluated the clinical efficacy of EASD and compared it to repeated RSD in Step 2 periodontal therapy and root surface re‐debridement in Step 3 periodontal therapy to manage periodontal pockets (Ardila and Vivares‐Builes 2023; Kuang et al. 2017). With the augmented subgingival vision, the EASD could improve the efficacy of calculus removal when compared to the closed debridement of the subgingival root surface. Simultaneously, the technically sensitive nature of the operation and the increased volume of information to manage during the periodontal endoscope‐assisted RSD procedure inevitably extend chairside time. This is especially noticeable when compared to repeated RSD procedures, especially during the Step 2 periodontal therapy. While periodontal endoscope has been shown to enhance calculus removal efficacy during repeated RSD in deep periodontal pockets, this does not necessary translate into clinical benefits for improving clinical parameters in nonspecific clinical scenarios. In the case of treating periodontitis during Step 2 therapy, the advantages of applying periodontal endoscope‐assisted RSD over repeated RSD may not be readily apparent due to different degrees of responses at sites with different characteristics. The clinical benefit of periodontal endoscope‐assisted RSD is particularly evident when addressing moderate to deep pockets, but not at sites with shallow PPD (Liao et al. 2016; Naicker et al. 2022; Wu et al. 2022).

The two systematic reviews included studies that evaluated the application of the periodontal endoscope during RSD for managing residual periodontal defects as part of Step 3 and for managing mixed periodontal defects during Step 2 periodontal therapy. The absence of clearly defined clinical scenarios in the reviews' focus questions hinders the interpretation of the available evidence regarding benefits conferred by the endoscope. Moreover, the reviews reached conflicting conclusions on its application in NSPT. This discrepancy is likely due to heterogeneity among the included studies and the lack of a well‐formulated, focused research question addressing the specific indications for periodontal endoscope use.

The current systematic review focused on assessing the treatment outcomes of residual periodontal defects after EASD, which did not respond fully to initial Step 1 and 2 periodontal therapy. These specific defects represent a clinical scenario where the efficacy of traditional RSD may be limited and thereby necessitating further surgical intervention in periodontal care in usual circumstances. Repeated RSD was demonstrated to produce limited clinical efficacy in resolving residual periodontal defects in the current literature (Tomasi et al. 2008; Wennström et al. 2001). The standard of care to manage moderate to deep periodontal defects is periodontal surgical approaches, including access flap, regenerative and resective approaches, according to various clinical situations (Sanz et al. 2020). The current systematic review focuses on evaluating the treatment outcome of EASD to manage moderate to deep residual periodontal defects as part of Step 3 periodontal therapy and has exclusively included the studies that investigated the adjunctive effect of periodontal endoscopy to Step 3 periodontal therapy. It is expected that more information could aid treatment decisions regarding the use of periodontal endoscope‐assisted RSD, conventional root surface re‐debridement, and periodontal surgery for managing residual periodontal defects in Step 3 periodontal therapy.

Since repeated RSD as in Step 3 periodontal therapy to manage moderately deep to deep residual periodontal defects resulted in limited improvement on periodontal pocket resolution (Tomasi et al. 2008; Wennström et al. 2001), it is important to understand whether periodontal endoscopy can bring additional benefit to the existing Step 3 periodontal therapies. The current systematic review demonstrated that EASD can result in an significant improved PPD reduction with WMD 0.84 mm and a significant improved gain in CAL with WMD 0.89 mm in favor of EASD when compared to repeated RSD at 6‐month postoperation. Comparing to the systematic review, which compared the clinical outcome of surgical and NSPT, it was reported that surgical periodontal therapy resulted in 0.6 mm more PPD reduction and 0.2 mm more gain in CAL in sites with PPD > 6 mm, and 0.4 mm more PPD reduction and 0.4 mm more CAL at sites with PPD 4–6 mm when compared to NSPT (Heitz‐Mayfield et al. 2002). The clinical benefits of EASD over repeated RSD appear to be comparable to the clinical benefits achieved by surgical periodontal therapy over NSPT. This finding may support EASD as a treatment alternative to routine unassisted repeated subgingival debridement in selected cases to manage moderately deep to deep residual periodontal defects as part of Step 3 periodontal therapy.

In the comparison between EASD and AFPS, a WMD of 0.21 mm (95% CI: −0.49 to 0.92) more gain in CAL was reported in favor of EASD, notwithstanding a WMD of −0.29 mm (95% CI: −0.69 to 0.11) more reduction in PPD in favor of AFPS at 3 months postoperation; however, the difference did not reach a significant difference between the two approaches.

EASD's advantage may be attributed to its flapless approach, which induces less trauma to the surrounding tissue and provides a more stable wound environment for periodontal healing compared to AFPS. Conversely, the higher reduction in PPD in AFPS may be explained by the granulation tissue removal permitted by the open flap approach, which allows for better adaptation of soft tissue along the alveolar bone crest during postoperative tissue remodeling.

It is noteworthy that the WMD in the reduction of PPD between EASD and repeated RSD was reported to be 0.84 and 0.50 mm, and the WMD in the gain of CAL between EASD and repeated RSD was reported to be 0.89 and 0.34 mm at 6 and 3 months postoperation, respectively. The difference for gain in CAL was statistically significant for 6 months but not 3 months, while the difference in reduction in PPD was statistically significant for both 3 and 6 months. It could possibly be explained that a longer time is required for healing by reattachment and bone remodeling than healing by recession and thus it takes longer duration for substantial change in CAL to be detected. This aligns with the current knowledge of periodontal healing patterns following nonsurgical periodontal treatment (Badersten et al. 1981, 1984) and is corroborated by a clinical trial comparing EASD versus repeated RSD in Step 2 periodontal therapy (Naicker et al. 2022).

Despite the relatively short observation period, 2 out of the 5 included studies did not report a detailed protocol of randomization, and 3 studies did not report treatment assignment revelation procedure in their reports. The unclear assignment concealment policy impacted negatively on the risk of bias assessment of the included studies. Due to the nature of the application of the periodontal endoscope, it made masking of patients and operators impossible. The assignment should be masked until as close as the time of operation delivery if possible. All 5 studies had applied an independent blinded assessor to carry out the measurement of clinical parameters to minimize potential bias. Nevertheless, studies should clearly state their caregiver masking policy during the supportive periodontal care period. This is to ensure that the quality of follow‐up care remains consistent across patients receiving different treatments, thereby minimizing potential bias. However, only one study reported a specific protocol in this particular aspect (Ho et al. 2025).

The current review offers insights into the clinical efficacy of EASD in comparison to traditional RSD and AFPS in managing residual periodontal pockets during Step 3 periodontal treatment. According to the CPG for Stage 1–3 Periodontitis (Sanz et al. 2020), periodontal surgery with a regenerative approach can be considered for the defects with intrabony components ≥ 3 mm. Multiple studies have documented that minimally invasive periodontal surgical regenerative approaches can result in improved treatment outcomes to manage such clinical scenarios (Cortellini and Tonetti 2009, 2011; Trombelli et al. 2012). Attempts had also been made to perform periodontal regeneration after nonsurgical periodontal treatment (Graziani et al. 2019; Wennström and Lindhe 2002).

Nonsurgical periodontal treatment has several limitations that making it less effective in managing deep periodontal pockets when compared to the periodontal surgical approach (Heitz‐Mayfield et al. 2002). Operators often rely on their tactile sensation to determine the endpoint of the subgingival instrumentation, but yet, complete removal of calculus is frequently unattainable. While this systematic review demonstrated the noninferiority of EASD to ASPF, it may inform better‐designed randomized clinical controlled trials in the future to evaluate the adjunct use of EASD in other flapless periodontal operations (i.e., periodontal regeneration).

It is worth noting that in two of the included studies in this systematic review, the control groups presented with significantly higher plaque indices compared to the groups treated with EASD (Pei et al. 2023; Wu et al. 2022). This imbalance may introduce a potential bias, as the poorer oral hygiene status in the control groups could have negatively impacted their clinical outcomes, thereby exaggerating the apparent effectiveness of endoscopic‐assisted therapy. Moreover, in four out of the five included studies, a range of tooth types, i.e. single‐rooted and multi‐rooted teeth, were included, with varying local risk factors, such as furcation involvement, dimensions of entry and exit of functions. Length of furcation trunk, dimensions of infra‐bony defects, type of teeth treated, etc., that may have an impact on the therapeutic outcomes of different treatment modalities. Due to the limited availability of the reported data, all clinical outcomes were pooled for analysis in the current systematic review. This approach, while necessary, may have introduced heterogeneity and limited the ability to evaluate treatment effects across different tooth types or risk profiles. It should be carefully considered when interpreting the overall results of the present systematic review. Future studies should consider stratified analyses or standardized reporting to facilitate more nuanced subgroup comparisons.

EASD was found to be achieving higher prevalence ratio of pocket resolution, 1.20 (95% CI: 1.03–1.40) when compared to repeated RSD at 6 month postoperation. It is worth noting that most of the included studies in this review are cluster design RCTs, and 0.4 ICC was applied to adjust for the patient clustering effect in all the analysis in the current study. A sensitivity analysis with an ICC assumption of 0.2 was performed. While most of the results remained unchanged, the difference in the prevalence ratio of pocket resolution at 3 months postoperation in the comparison between EASD versus repeated RSD was changed from insignificance to significance (*p* = 0.126, ICC = 0.4; *p* = 0.035, ICC = 0.2). Therefore, interpretation of this result needs to be in caution due to uncertainty in the degree of clustering of the included studies. Further studies with precise ICC estimates are needed to confirm the effect.

Another limitation in the current systematic review was the scarcity of studies reporting on patient‐reported outcome measures (PROMs) and radiographic outcomes after EASD. Only one study provided data on these crucial aspects of treatment success (Ho et al. 2025). This highlights a significant gap in the existing research and underscores the need for future investigations to comprehensively evaluate the patient experience and radiographic responses following EASD.

On comparing EASD and repeated RSD for managing residual periodontal pockets, the GRADE assessment rated the quality of the evidence to be “low,” and the comparison between EASD and AFPS was rated “moderate.” Future research should focus on well‐designed, bias‐controlled RCTs to evaluate the clinical efficacy of EASD as part of Step 3 periodontal therapy.

## 5 Conclusions

According to this systematic review for the management of moderately deep and deep residual periodontal defects in Step 3 periodontal therapy, EASD resulted in 0.5 mm greater reduction in PPD at 3‐month postoperation; a 0.84 mm greater reduction in PPD and a 0.89 mm greater gain in CAL at 6‐months postoperation, respectively, compared to repeated RSD. A significantly higher prevalence ratio (1.20) of pocket resolution in favor of EASD over repeated RSD was observed at 6 months postoperatively.

When comparing EASD to AFPS, no significant difference in PPD reduction, CAL gain and prevalence ratio of pocket resolution were observed at 3‐month postoperation follow‐up.

The overall certainty of the evidence was deemed to be “low” for EASD versus repeated RSD comparisons and “moderate” for EASD versus AFPS comparisons. Further high‐quality research is necessary to validate the clinical benefits of using a periodontal endoscope in Step 3 periodontal therapy.

## Author Contributions

King‐Lun Dominic Ho and Melissa Rachel Fok contributed to study design, protocol development, methodology, experimental work, data curation, analysis, and contributed equally to this study. Georgios Pelekos contributed to methodology, resources, and supervision. Kar Yan Li contributed to data curation and analysis. Wai Keung Leung contributed to methodology, resources, supervision, and project administration. All authors contributed to data interpretation and manuscript preparation.

## Conflicts of Interest

The authors declare that no potential conflict of interest. The research was solely supported by the Faculty of Dentistry, the University of Hong Kong, and the Health and Medical Research Fund of Hong Kong (grant number 18170492).

## Supporting information


**Supplementary Figure S1:** Cochrane risk‐of‐bias for randomized trials (RoB2) assessment concerning the included studies. **Supplementary Figure S2:** Mean difference of clinical attachment level (CAL) 3‐month post‐treatment of endoscope‐assisted subgingival debridement (EASD) or access flap periodontal surgery (AFPS). **Supplementary Figure S3:** Mean difference of probing pocket depth (PPD) 3‐month post‐treatment of endoscope‐assisted subgingival debridement (EASD) or access flap periodontal surgery (AFPS). **Supplementary Figure S4:** Mean difference of recession 3‐, 6‐, 9‐ or 12‐month post‐treatment of endoscope‐assisted subgingival debridement (EASD) or access flap periodontal surgery (AFPS). Results from only one study (Ho, Ho, Pelekos, Leung, & Tonetti, 2025). **Supplementary Figure S5:** Prevalence ratio of pocket resolution (PPD ≤ 4 mm) 3‐month post‐treatment of endoscope‐assisted subgingival debridement (EASD) or access flap periodontal surgery (AFPS). **Supplementary Figure S6:** Grade assessment on evidence comparing endoscope‐assisted subgingival debridement (EASD) or repeated root surface debridement (RSD). **Supplementary Table S1:** Excluded studies.

## Data Availability

The data that support the findings of this study are available from the corresponding author upon reasonable request.
