# Supplementary material for: Effectiveness of Endoscope‐Assisted Subgingival Debridement Versus Repeated Root Surface Debridement or Access Flap Periodontal Surgery in Step 3 Periodontal Therapy: A Systematic Review and Meta‐Analysis
Source: Clin Exp Dent Res. 2025 Jul 31;11(4):e70196. doi: 10.1002/cre2.70196 (PMC12312801; doi:10.1002/cre2.70196)
Supplement: Supplementary file 1 — Supplementary Figure S1: Cochrane risk‐of‐bias for randomized trials (RoB2) assessment concerning the included studies. Supplementary Figure S2: Mean difference of clinical attachment level (CAL) 3‐month post‐treatment of endoscope‐assisted subgingival debridement (EASD) or access flap periodontal surgery (AFPS). Supplementary Figure S3: Mean difference of probing pocket depth (PPD) 3‐month post‐treatment of endoscope‐assisted subgingival debridement (EASD) or access flap periodontal surgery (AFPS). Supplementary Figure S4: Mean difference of recession 3‐, 6‐, 9‐ or 12‐month post‐treatment of endoscope‐assisted subgingival debridement (EASD) or access flap periodontal surgery (AFPS). Results from only one study (Ho, Ho, Pelekos, Leung, & Tonetti, 2025). Supplementary Figure S5: Prevalence ratio of pocket resolution (PPD ≤ 4 mm) 3‐month post‐treatment of endoscope‐assisted subgingival debridement (EASD) or access flap periodontal surgery (AFPS). Supplementary Figure S6: Grade assessment on evidence comparing endoscope‐assisted subgingival debridement (EASD) or repeated root surface debridement (RSD). Supplementary Table S1: Excluded studies. [file CRE2-11-e70196-s001.docx]

**SUPPLMENTARY INFORMATION**


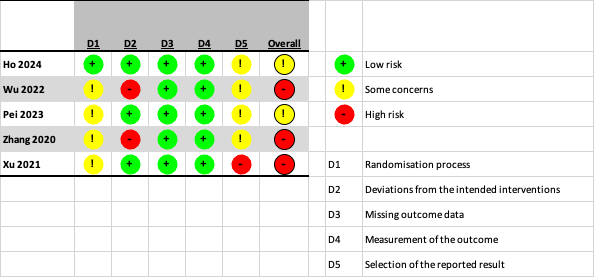


**SUPPLMENTARY FIGURE 1** Cochrane risk-of-bias for randomized trials (RoB2) assessment concerning the included studies.

**SUPPLMENTARY FIGURE 2** Mean difference of clinical attachment level (CAL) 3-month post-treatment of endoscope-assisted subgingival debridement (EASD) or access flap periodontal surgery (AFPS).

**SUPPLMENTARY FIGURE 3** Mean difference of probing pocket depth (PPD) 3-month post-treatment of endoscope-assisted subgingival debridement (EASD) or access flap periodontal surgery (AFPS).

**SUPPLMENTARY FIGURE 4** Mean difference of recession 3-, 6-, 9- or 12-month post-treatment of endoscope-assisted subgingival debridement (EASD) or access flap periodontal surgery (AFPS). Results from only one study (Ho, Ho, Pelekos, Leung, & Tonetti, 2025).

**SUPPLMENTARY FIGURE 5** Prevalence ratio of pocket resolution (PPD ≤4mm) 3-month post-treatment of endoscope-assisted subgingival debridement (EASD) or access flap periodontal surgery (AFPS).


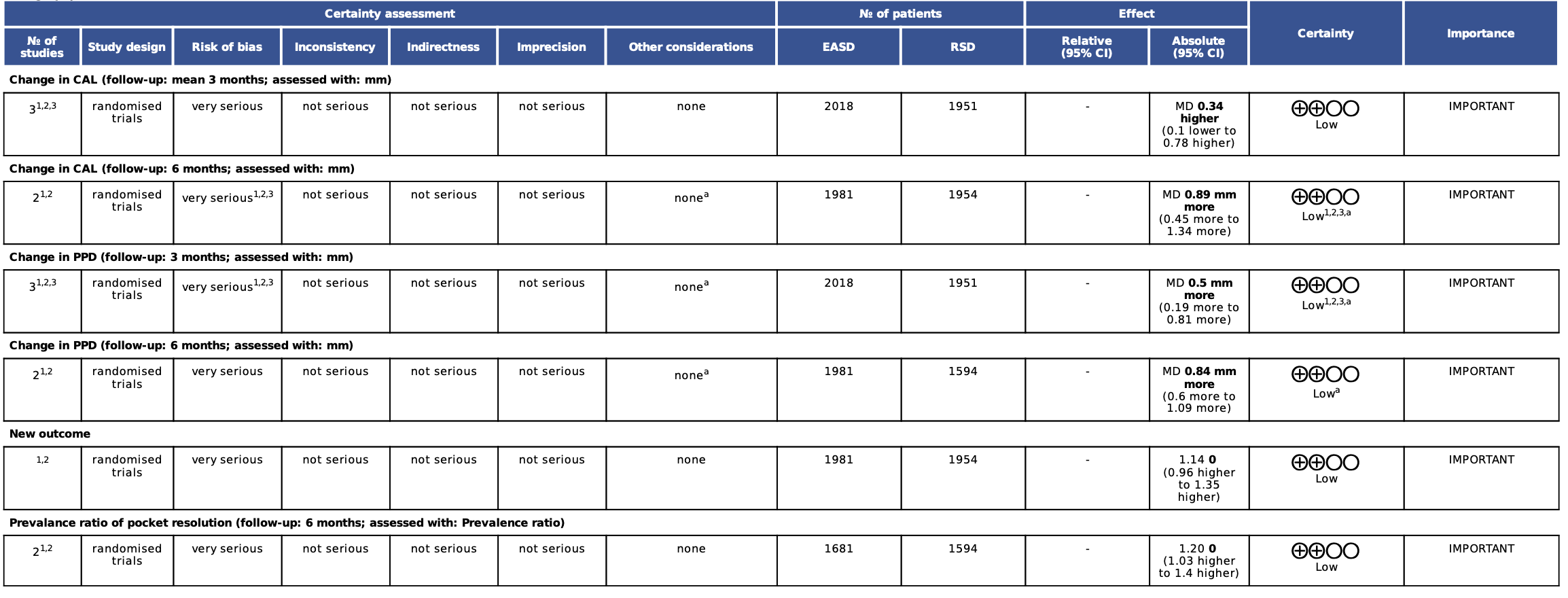


**SUPPLMENTARY FIGURE 6** Grade assessment on evidence comparing endoscope-assisted subgingival debridement (EASD) or repeated root surface debridement (RSD).

**SUPPLEMENTARY TABLE 1** Excluded studies

| Number | Reference | Reason for exclusion |
| --- | --- | --- |
| 1 | Avradopoulos, Wilder, Chichester, and Offenbacher (2004) | Wrong Outcome: Not reporting data in this systematic review interest |
| 2 | Blue, Lenton, Lunos, Poppe, and Osborn (2013) | Wrong indication: Application of Periodontal endoscope in Step 2 periodontal therapy |
| 3 | Song, Kuang, Hu, Chen, and Feng (2018) | Wrong study design: Journal Correspondence |
| 4 | Stambaugh (2000) | Wrong study design: Conference abstract. No full text available. |
| 5 | Stephen K. Harrel (2014) | Wrong study design: Book Chapter |
| 6 | S. K. Harrel, Abraham, Rivera-Hidalgo, Shulman, and Nunn (2014) | Wrong study design: non-RCT |
| 7 | S. K. Harrel, Abraham, Rivera-Hidalgo, Shulman, and Nunn (2016) | Wrong study design: non-RCT |
| 8 | S. K. Harrel et al. (2017) | Wrong study design: non-RCT |
| 9 | Su, Wang, Zhang, and Wu (2024) | Exclusion criteria: self-control pretest-posttest study. |
| 10 | J. H. Shi et al. (2020) | Wrong intervention: A RCT comparing periodontal endoscopy-aided non-incisional regeneration technique versus periodontal endoscopy-aided scaling and root planing |
| 11 | J. Shi et al. (2023) | Wrong study design: non-RCT |
| 12 | J. H. Shi, Li, Jiang, Lei, and Li (2024) | Exclusion criteria: non-RCT |
| 13 | Lu Jingyi (2019) | Wrong study design: case reports |
| 14 | Xia and Li (2021) | Wrong indication: Application of Periodontal endoscope in Step 2 periodontal therapy |
| 15 | Yang, Wang, Lei, and Li (2024) | Wrong indication: Application of Periodontal endoscope in Step 2 periodontal therapy |
| 16 | Zhao, Tan, Li, Zhang, and Chen (2021) | Wrong indication: Application of Periodontal endoscope in Step 2 periodontal therapy |

**REFERENCE**

Avradopoulos, V., Wilder, R. S., Chichester, S., & Offenbacher, S. (2004). Clinical and inflammatory evaluation of Perioscopy on patients with chronic periodontitis. *J Dent Hyg, 78*(1), 30-38.

Blue, C. M., Lenton, P., Lunos, S., Poppe, K., & Osborn, J. (2013). A pilot study comparing the outcome of scaling/root planing with and without Perioscope technology. *J Dent Hyg, 87*(3), 152-157.

Harrel, S. K. (2014). The MIS and V-MIS Surgical Procedure. In T. G. W. J. Stephen K. Harrel (Ed.), *Minimally Invasive Periodontal Therapy: Clinical Techniques and Visualization Technology* (pp. 81-116): John Wiley & Sons, Inc.

Harrel, S. K., Abraham, C. M., Rivera-Hidalgo, F., Shulman, J. D., & Nunn, M. E. (2014). Videoscope-assisted minimally invasive periodontal surgery (V-MIS). *J Clin Periodontol, 41*(9), 900-907. doi:10.1111/jcpe.12294

Harrel, S. K., Abraham, C. M., Rivera-Hidalgo, F., Shulman, J. D., & Nunn, M. E. (2016). Videoscope-Assisted Minimally Invasive Periodontal Surgery: One-Year Outcome and Patient Morbidity. *Int J Periodontics Restorative Dent, 36*(3), 363-371. doi:10.11607/prd.2759

Harrel, S. K., Nunn, M. E., Abraham, C. M., Rivera-Hidalgo, F., Shulman, J. D., & Tunnell, J. C. (2017). Videoscope Assisted Minimally Invasive Surgery (VMIS): 36-Month Results. *J Periodontol, 88*(6), 528-535. doi:10.1902/jop.2017.160705

Ho, K. D., Ho, K. R., Pelekos, G., Leung, W. K., & Tonetti, M. S. (2025). Endoscopic Re-Instrumentation of Intrabony Defect-Associated Deep Residual Periodontal Pockets Is Non-Inferior to Papilla Preservation Flap Surgery: A Randomized Trial. *J Clin Periodontol, 52*(2), 289-298. doi:10.1111/jcpe.14075

Lu Jingyi, W. L., Zheng Yi, Ren Baijie, Wang Xiaojun. (2019). Clinical application of non-surgical treatment under periodontal endoscope in patients with moderate-to- severe periodontitisof multirooted teeth. *Journal of Jilin University (MEdicine Edition), 45*(5), 1146-1151. doi:10.13481/j.1671-587x.20190529

Shi, J., Wang, J., Yang, Z., Li, J., Lei, L., & Li, H. (2023). A novel periodontal endoscopy-aided non-incisional periodontal regeneration technique in the treatment of intrabony defects: a retrospective cohort study. *BMC Oral Health, 23*(1), 962. doi:10.1186/s12903-023-03674-9

Shi, J. H., Li, J. W., Jiang, L. S., Lei, L., & Li, H. X. (2024). [A novel periodontal endoscopy-aided non-incisional periodontal regeneration technique：a case series study]. *Shanghai Kou Qiang Yi Xue, 33*(1), 80-84.

Shi, J. H., Xia, J. J., Lei, L., Jiang, S., Gong, H. C., Zhang, Y., . . . Li, H. X. (2020). [Efficacy of periodontal endoscope-assisted non-surgical treatment for severe and generalized periodontitis]. *Hua Xi Kou Qiang Yi Xue Za Zhi, 38*(4), 393-397. doi:10.7518/hxkq.2020.04.007

Song, J., Kuang, Y., Hu, B., Chen, J., & Feng, G. (2018). Authors’ response. *The Journal of the American Dental Association, 149*(3), 170-171. doi:10.1016/j.adaj.2018.01.021

Stambaugh, R. M., GC; Watanabe, J; Lass, C; Stambaugh, KA (2000). Abstracts of Papers: Endoscopic instrumentation of the subgingival root surface in periodontal therapy. *JOURNAL OF DENTAL RESEARCH, 79*, 489-489. doi:10.1177/0022034500079S108

Su, Q., Wang, N., Zhang, M., & Wu, J. (2024). Clinical efficacy of periodontal endoscopy-assisted subgingival scaling and root planning and its effect on psychology and quality of life in patients with periodontitis. *Journal of Prevention and Treatment for Stomatological Diseases, 32*(1), 50-56. doi:10.12016/j.issn.2096-1456.2024.01.008

Xia, J., & Li, H. (2021). Clinical efficacy evaluation of minimally invasive periodontal therapy assisted by endoscopy. *Journal of Prevention and Treatment for Stomatological Diseases, 29*(3), 171-177. doi:<https://doi.org/10.12016/j.issn.2096-1456.2021.03.005>

Yang, Z., Wang, J., Lei, L., & Li, H. (2024). Two-year follow-up of the outcomes of endoscope-assisted minimally invasive nonsurgical periodontal therapy for deep intrabony defects. *Journal of Prevention and Treatment for Stomatological Diseases, 32*(5), 350-358. doi:10.12016/j.issn.2096-1456.2024.05.004

Zhao, J., Tan, B., Li, L., Zhang, Y., & Chen, S. (2021). Effects of ultrasonic subgingival scaling and root planing with a periodontal endoscope on the root surface. *Journal of Prevention and Treatment for Stomatological Diseases, 29*(10), 684-688. doi:<https://doi.org/10.12016/j.issn.2096-1456.2021.10.006>
